# Monitoring the Activity and Inhibition of Cholinesterase Enzymes using Single-Walled Carbon Nanotube Fluorescent Sensors

**DOI:** 10.1021/acs.analchem.2c02471

**Published:** 2022-10-07

**Authors:** Dan Loewenthal, Dotan Kamber, Gili Bisker

**Affiliations:** † School of Chemistry, Faculty of Exact Sciences, Tel-Aviv University, Tel Aviv6997801, Israel; ‡ Department of Analytical Chemistry, Israel Institute for Biological Research, Ness-Ziona7410001, Israel; § Department of Biomedical Engineering, Faculty of Engineering, Tel-Aviv University, Tel Aviv6997801, Israel; ∥ The Center for Physics and Chemistry of Living Systems, Tel-Aviv University, Tel Aviv6997801, Israel; ⊥ Center for Nanoscience and Nanotechnology, Tel-Aviv University, Tel Aviv6997801, Israel; # Center for Light Matter Interaction, Tel-Aviv University, Tel Aviv6997801, Israel

## Abstract

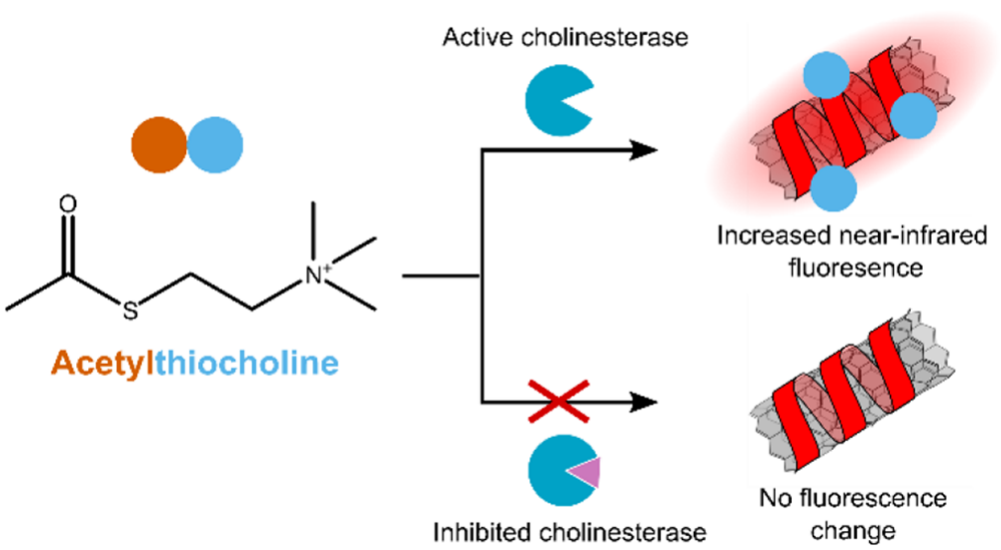

Cholinesterase enzymes are involved in a wide range of bodily functions, and their disruption is linked to pathologies such as neurodegenerative diseases and cancer. While cholinesterase inhibitors are used as drug treatments for diseases such as Alzheimer and dementia at therapeutic doses, acute exposure to high doses, found in pesticides and nerve agents, can be lethal. Therefore, measuring cholinesterase activity is important for numerous applications ranging from the search for novel treatments for neurodegenerative disorders to the on-site detection of potential health hazards. Here, we present the development of a near-infrared (near-IR) fluorescent single-walled carbon nanotube (SWCNT) optical sensor for cholinesterase activity and demonstrate the detection of both acetylcholinesterase and butyrylcholinesterase, as well as their inhibition. We show sub U L^–1^ sensitivity, demonstrate the optical response at the level of individual nanosensors, and showcase an optical signal output in the 900–1400 nm range, which overlaps with the biological transparency window. To the best of our knowledge, this is the longest wavelength cholinesterase activity sensor reported to date. Our near-IR fluorescence-based approach opens new avenues for spatiotemporal-resolved detection of cholinesterase activity, with numerous applications such as advancing the research of the cholinergic system, detecting on-site potential health hazards, and measuring biomarkers in real-time.

## Introduction

Cholinesterase (ChE) enzymes hydrolyze choline esters in the body and are linked with a diverse range of bodily functions and pathologies. The two major cholinesterase enzymes are Acetylcholinesterase (AChE) and butyrylcholinesterase (BChE).^1^ Acetylcholinesterase is found mainly at neuronal synapses, where it modulates neuronal transmission via hydrolysis of acetylcholine. Disruption of AChE has been linked with pathologies such as Alzheimer’s disease, Parkinson’s disease, Myasthenia gravis, depression, organophosphorus poisoning, and cancer.^2−4^ Butyrylcholinesterase is a less specific ChE enzyme, found mainly in the plasma, and its disruption has been linked with sudden infant death syndrome and Alzheimer’s disease, potentially serving as a biomarker.^5^

While cholinesterase inhibitors can serve as therapeutics, high doses found in organophosphorus pesticides and nerve agents cause cognitive decline and mortality.^6^ Therefore, measuring the activity of these enzymes is important for the detection of health hazards, biomarker monitoring, studies of the cholinergic system, and the search for novel therapeutics for neurodegenerative diseases.

Traditionally, ChE activity is measured by the Ellman absorbance-based assay,^7^ in which acetylthiocholine (ATC), a synthetic analog of acetylcholine, is hydrolyzed by a cholinesterase enzyme to produce thiocholine, which subsequently reacts with 5,5′-dithiobis(2-nitrobenzoic acid) to produce a yellow color, which can be measured using absorbance spectroscopy. This approach, however, only measures average quantities (bulk).

Over the past few years, many research efforts focused on developing fluorescent nanosensors for ChE activity and inhibition,^8−16^ some of which were able to provide spatiotemporal resolution.^9−11,14^ Fluorescent sensors hold promise for superior optical selectivity over the more traditional absorbance-based methods when measuring in the presence of biological media,^7,17^ while spatiotemporal-resolving sensors offer exciting possibilities over the more traditional bulk measurements. For example, such spatiotemporal information can potentially serve as tumor resection guides due to cholinesterase activity difference between brain glioma cells and normal brain cells.^9^ However, many of these new methods involve complex cascade reactions or experimental setups, and their optical outputs are usually up to a wavelength of 800 nm at most, limiting tissue penetration and the signal-to-noise ratio due to tissue autofluorescence.^18,19^

Single-walled carbon nanotubes (SWCNTs) benefit from unique optical and electronic properties, which render them favorable fluorescent probes for imaging, sensing, and biomedical applications,^20−26^ owing to their fluorescence in the near-IR range where tissue, blood, and biological samples in general are mostly transparent.^27−34^ Moreover, SWCNT sensors are stable at room temperature, provide spatiotemporal information, and do not photobleach upon use, unlike many other fluorescent sensors.^35−37^ The mechanism of SWCNT-based sensors usually relies on tailored functionalization of the nanotube surface, which mediates the interaction with the analyte of interest, such that binding of the target molecule results in a modulation of the emitted fluorescence.^38−41^ Fluorescent SWCNT sensors were applied for the biosensing of different analytes and enzymes.^23,29,31,37,42−50^ These range from monitoring progesterone and cortisol in vivo (mice),^31^ fibrinogen and insulin in blood and cell culture,^45,48^ nitroaromatics^29^ and pathogens^49,51^ in vivo (plants), volatiles in the gaseous phase,^52^ to enzymatic activity.^53−55^

**Scheme 1:**
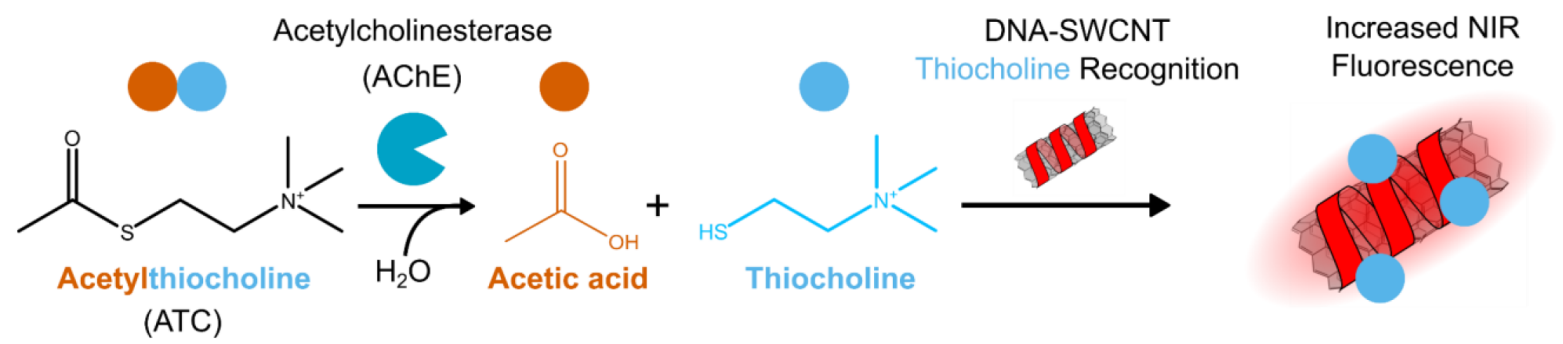
AChE Activity Sensing Mechanism AChE hydrolyses acetylthiocholine to acetic acid and thiocholine; thiocholine is then recognized by a DNA-SWCNT sensor resulting in increased near-IR fluorescence intensity.

Here, we present a sensitive, near-IR, SWCNT fluorescent nanosensor for ChE activity and inhibition. The sensor is based on selective recognition of thiocholine, the cholinesterase-hydrolysis product of acetylthiocholine, triggering a near-IR fluorescence intensity increase of DNA-functionalized SWCNTs (Scheme 1). We elucidate the recognition mechanism, demonstrate an optical output in the 900–1400 nm range, find a sub U L^–1^ limit of detection, demonstrate the ability to infer BChE inhibition, and last, resolve the fluorescence response to thiocholine at the single-nanosensor level with spatiotemporal information.

## Experimental Section

### Materials

HipCO SWCNTs were purchased from Nanointegris. DNA oligomers, (GT)_15_, (TAT)_4_, (GTTT)_7_ (GC)_30_, (T)_30_, and (GT)_15_, were purchased from Integrated DNA Technologies. Recombinant human AChE and BChE were supplied by the Israel Institute for Biological Research. l-Cysteine and neostigmine bromide were purchased from Holland Moran (Israel). Acetic acid, choline chloride, thiocholine iodide, 5,5-dithio-bis(2-nitrobenzoic acid) (DTNB), and 0.1% poly-l-lysine were purchased from Sigma-Aldrich (Israel).

### DNA-SWCNTs Suspension

A total of 1 mg of HipCO SWCNTs and 2 mg of DNA in 0.1 M NaCl were bath sonicated (Elma P-30H) for 10 min at 80 Hz and tip-sonicated twice (Qsonice Q125, 3 mm tip, 4 W) for 20 min in an ice bath. The suspension was centrifuged twice at 16100 rcf for 90 min, where after each centrifugation step, 80% of the supernatant was collected and the rest was discarded. The resulting DNA-SWCNT concentration was measured by absorbance spectroscopy (Shimadzu UV-3600 Plus)^35,56^ using an extinction coefficient^35^ of ε_632 nm_ = 0.036 L mg^–1^ cm^–1^.

The DNA oligomers screened were (5′ to 3′): (GT)_15_, (TAT)_4_, (GTTT)_7_ (GC)_30_, and (T)_30_.

### Near-IR Fluorescence Spectroscopy of DNA-SWCNTs

The fluorescence intensity of the DNA-SWCNT samples was measured on a near-IR inverted fluorescence microscope (Olympus IX73). A 730 nm continuous-wave laser (MDL-MD-730–1.5W, Changchun New Industries) was used for excitation and a near-IR spectrometer (Spectra Pro HRS-300, Princeton Instruments) with a slit-width of 500 μm and a grating of 150 g/mm coupled to an InGaAs line detector (PylonIR, Teledyne Princeton Instruments) was used for spectrally resolved fluorescence detection, with a 1 s exposure time.

### DNA-SWCNTs Excitation–Emission Spectra

DNA-SWCNTs were diluted to 1 mg L^–1^ in PBS pH 7.4 and were illuminated with a supercontinuum white-light laser (NKT-photonics, Super-K Extreme) with a bandwidth filter (NKT-photonics, Varia, Δλ = 20 nm) scanned between 500 and 840 nm with an excitation time of 2 s per wavelength, a 1 nm wavelength step size, and 20 mW (at 730 nm) intensity. Emission spectra were recorded using the near-IR spectrometer described above. Spectra were background subtracted of a phosphate buffered saline (PBS, pH 7.4) sample and can be found in the Supporting Information (Figure S1).

### Screening DNA-SWCNT Library for AChE-Responsive Sensors

For each sensor, 147 μL of 1 mg L^–1^ DNA-SWCNT suspension in PBS was spiked with 3 μL of 5 mM acetylthiocholine (0.1 mM acetylthiocholine in the resulting solution) or 3 μL of PBS as control, and the fluorescence response was recorded after 1 h. Subsequently, samples that contained acetylthiocholine were spiked with AChE (3 μL, 10 U L^–1^ in the resulting solution), and the fluorescence response was recorded after 1 h. Data are presented as the normalized fluorescence intensity of the (9,4) DNA-SWCNT chirality.

### DNA-SWCNT Fluorescence Response Kinetics and Limit of Detection for AChE\BChE

A total of 147 μL of SWCNTs solution (1 mg L^–1^ in PBS pH 7.4) was spiked with 3 μL of 5 mM acetylthiocholine and incubated for 10 min to ensure stabilization. Afterward, 3 μL of the enzymes at different U L^–1^ activity were spiked to the SWCNTs solution, and the fluorescence spectra dynamics were monitored for 2 h with the near-IR inverted fluorescence microscope. The limit of detection (LOD) was calculated as the concentration at which the signal is three times the standard deviation of the noise level. Data are presented as the normalized fluorescence intensity of the (9,4) DNA-SWCNT chirality. Enzyme quantity is presented in U, which is the amount of enzyme that hydrolyses 1 μM of acetylthiocholine per minute at room temperature and pH 7.5. All error bars represent the standard deviation of experimental replicates.

### Sensor Kinetics and Concentration Dependence Response to Relevant Small Molecules

A total of 147 μL of SWCNTs solution (1 mg L^–1^ in PBS pH 7.4) was spiked with 3 μL of cysteine, acetylthiocholine, choline, and acetic acid for final concentrations of 10, 100, 300, and 1000 μM. A total of 3 μL of neostigmine was spiked for final concentrations of 1, 10, 30, and 100 μM. Data are presented as the normalized fluorescence intensity of the (9,4) DNA-SWCNT chirality. Samples were measured in triplicate and the fluorescence intensity was monitored with time. Signals were normalized to the first time point.

### BChE Inhibition Assay

A total of 147 μL of DNA-SWCNTs solutions (1 mg L^–1^ in PBS pH 7.4) was spiked with 3 μL of neostigmine (100 mM) and 3 μL of 0.5 U L^–1^ BChE solution in PBS pH 7.4 and stirred gently at room temperature for 10 min for cholinesterase inhibition. Subsequently, 3 μL of acetylthiocholine (5 mM) were spiked for a final concentration of 100 μM in solution, and the fluorescence response was monitored for 1 h. Signals were normalized to the fluorescence intensity before the addition of acetylthiocholine. Data are presented as the normalized fluorescence intensity of the (9,4) DNA-SWCNT chirality.

### Ellman Assay

The assay is based on a well-established protocol.^17^ Briefly, diluted samples were incubated with 5,5-dithio-bis(2-nitrobenzoic acid) at 37 °C for 10 min, to which acetylthiocholine was added to a final concentration of 0.45 mM in solution. Immediately after acetylthiocholine addition, sample absorbance was recorded at 436 nm for 3 min using a plate reader (Fusion Optics Reader Platform SPARK). The resulting linear absorption over time curve is a direct indication of the amount of 3-carboxy-4-nitrobenzenethiolate (ε = 10.6 × 103 M^–1^ cm^–1^) anion formed, which itself is in direct proportion to the amount of thiocholine formed in solution and thus ChE activity.

### Cholinesterase Activity Measurement in Serum

Fetal bovine serum (Sigma) was diluted in PBS in the range of 1:30 to 1:300 and the cholinesterase activity of each dilution was quantified with the Ellman assay. (GT)_15_-SWCNTs were incubated at 1 mg L^–1^ in the diluted serum solution for two hours. A total of 147 μL of the (GT)_15_-SWCNT in serum solution were spiked with 3 μL acetylthiocholine (22.5 mM stock concentration) for a final concentration of 450 μM, and the fluorescence intensity was monitored for 12 min. Signals were normalized to the fluorescence intensity at the beginning of the assay and compared to control samples of the same serum dilution to which no acetylthiocholine was added (Figure S5). Data are presented as the background-subtracted, normalized, fluorescence intensity of the (9,4) SWCNT chirality.

### Fluorescence Imaging

For enzyme activity imaging, an 18 mm × 18 mm glass coverslip was rinsed with water and EtOH, and left to dry. A total of 300 μL of 0.01% poly-l-lysine solution in water was pipetted onto each slide. After 15 min, the slides were washed with water and dried with a stream of nitrogen. A total of 300 μL of 1 mg L^–1^ (GT)_15_-SWCNT solution in water was added to each well for 15 min incubation, followed by a washing step with water and drying with a stream of nitrogen. Coverslips were then adhered to a plastic slide with a built-in well (Chroma Technology Corp). A total of 300 μL of acetylthiocholine (450 μM) in PBS was added to the well. After 2 min, 5 μL of diluted BChE solution were spiked into the well, and the fluorescence response over time was recorded. For thiocholine imaging, 200 μL of PBS were added to the coverslip well, followed by 100 μL of thiocholine solution (1.35 mM) for a final concentration of 450 μM thiocholine, and the fluorescence intensity change upon addition was recorded.

Images were taken with an inverted fluorescence microscope (Olympus IX83) at 100× magnification (100× 1.3 NA, Plan FL objective), operated in highly inclined and laminated optical sheet (HiLo) microscopy mode,^57^ to minimize the illumination depth compared to epi-fluorescence. Fluorescent samples were excited with a 730 nm continuous-wave laser (MDL-MD-730–1.5W, Changchun New Industries) at approximately ∼440 mW (20 W mm^–2^), and the near-IR emission was imaged after a 900 nm long-pass emission filter (Chroma ET900lp) with a cooled InGaAs-camera (Raptor, Ninox-640 Vis-NIR). Videos were taken at a frame rate of 2 frames per second, with 200 ms exposure time.

The fluorescence time-trace of individual DNA-SWCNT nanosensors in response to thiocholine was extracted using ImageJ.^58^

## Results and Discussion

### DNA-SWCNT Library Response to AChE and Acetylthiocholine

**Figure 1:**
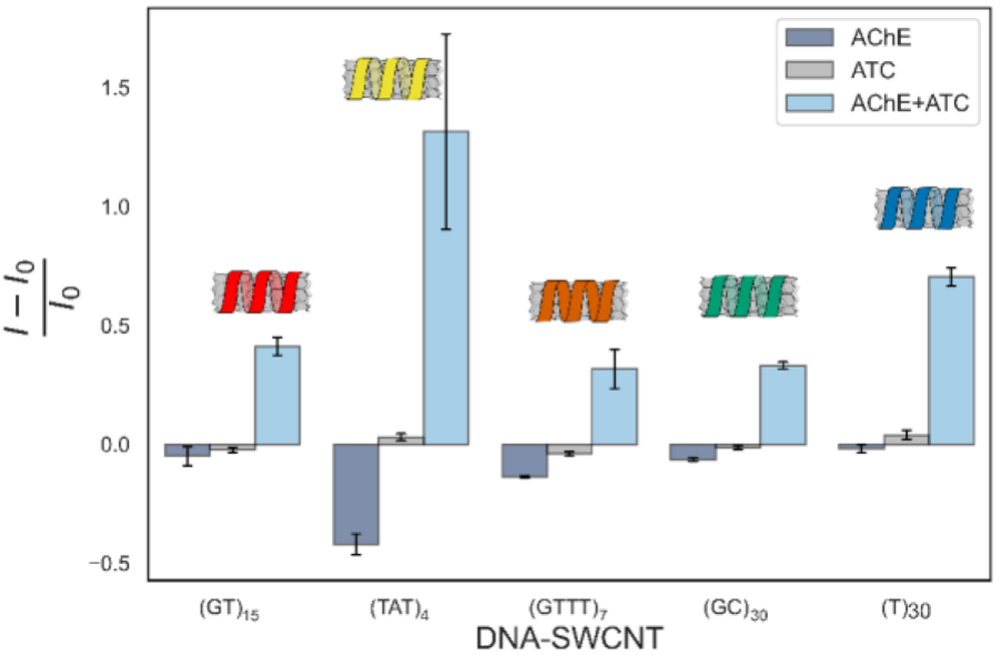
Normalized fluorescence response of different DNA-SWCNTs to either AChE, acetylthiocholine (ATC), or their combination (AChE + ATC). (GT)_15_-SWCNT and (T)_30_-SWCNT displayed a selective response toward the AChE+ATC combination. *I*_0_ and *I* are the initial and final fluorescence intensities, respectively. All error bars represent the standard deviation of experimental replicates (*n* = 3).

We chose a screening assay approach, based on previous successful demonstrations of SWCNT-sensors discovery, to find an optimal cholinesterase SWCNT-based sensor. In this approach, a library of DNA-SWCNTs are exposed to various analytes, establishing the specificity and sensitivity of these suspensions toward the selected analytes.^31,35,45,59,60^ To this end, we measured the fluorescence response of a library of DNA-SWCNTs, including (GT)_15_-, (T)_30_-, (TAT)_4_-, (GTTT)_7_-, and (GC)_30_-SWCNT, to either AChE, its substrate acetylthiocholine (ATC), or their combination (Figure 1). The rational was to identify a DNA-SWCNT suspension that would selectively respond to the byproducts of the hydrolytic reaction between AChE and ATC, but would be inactive to each of the initial reactants.

(GT)_15_-SWCNTs and (T)_30_-SWCNTs exhibited significant fluorescence increase of ∼50% and ∼70%, respectively, of the (9,4) chirality, when exposed to the AChE+ATC combination, whereas the exposure to either AChE or ATC alone resulted in a negligible response. As such, they were chosen for further investigation. (TAT)_4_-SWCNTs also exhibited a large response toward the AChE+ATC combination, however, it was not selective and responded significantly to AChE alone. (GC)_30_-SWCNTs showed a selective but smaller response compared to (GT)_15_-SWCNTs and (T)_30_-SWCNTs, and the response of (GTTT)_7_-SWCNTs was relatively small and less selective (Figure S2).

### Elucidating the Sensing Mechanism through the Response to Relevant Small Molecules

We exposed the sensors to varying concentrations of relevant small molecules to elucidate the exact chemical characteristics responsible for the fluorescence response of the DNA-SWCNTs during AChE enzymatic activity (Figure S3). We measured the fluorescence response toward, acetylthiocholine, the hydrolysis substrate, acetic acid and thiocholine, the hydrolysis products, as well as cysteine and choline to further probe the chemical functional groups responsible for the fluorescence response of the DNA-SWCNT sensors. We also measured the response toward an AChE inhibitor, neostigmine, at relevant concentrations.

We found a concentration-dependent significant fluorescence intensity response for both (GT)_15_-SWCNTs and (T)_30_-SWCNTs to thiocholine. The rest of the tested molecules induced small to negligible responses. Previously developed AChE-sensors relied on targeting or recognizing the thiol group present in thiocholine,^7,16,61^ which may partly contribute to the fluorescence response in our case. To test this option, we monitored the response to cysteine, a thiol-containing small molecule, and found an ∼25% fluorescence increase at the relevant thiol concentrations for (T)_30_-SWCNTs and an ∼35% fluorescence increase for (GT)_15_-SWCNTs (Figure S3). These values are smaller than expected if the mechanism was a simple recognition of thiols, in which case the response to thiols would be similar to the response to thiocholine. Therefore, the sensing cannot be explained by recognition of the thiol group, but rather a more complex mechanism governs the interaction and the resulting fluorescence response. Possible mechanisms include the direct interaction of thiocholine with the DNA corona or the SWCNT surface, conformational change of the DNA, changes in the SWCNT surface accessibility to water molecules or specific functional groups, and modulations in the solvent or ion distribution in close proximity to the nanotube surface upon introduction of thiocholine.^23,62−64^ All the above mechanisms can significantly affect the DNA-SWCNT exciton properties and modulate the fluorescence intensity observed.^23,62^ One of the most plausible contributing mechanisms, in this case, is a perturbation of the DNA corona, as was demonstrated both experimentally and computationally for a similar system.^35,65^ The fluorescence response of (GT)_15_-SWCNTs and (T)_30_-SWCNTs to acetylthiocholine at large concentrations might be attributed to spontaneous hydrolysis of acetylthiocholine to thiocholine during the assay.

### Sensor Calibration with Different Concentrations of AChE and BChE

As the DNA-SWCNT sensors detect thiocholine, the sensors are also suitable for detecting ChE activity. Thus, we monitored the fluorescence response of the DNA-SWCNT sensors to different concentrations of both AChE and BChE between 10^–4^ and 10^2^ U L^–1^ well within the relevant window for characterizing enzymatic activity and inhibition in the body.^66^

**Figure 2:**
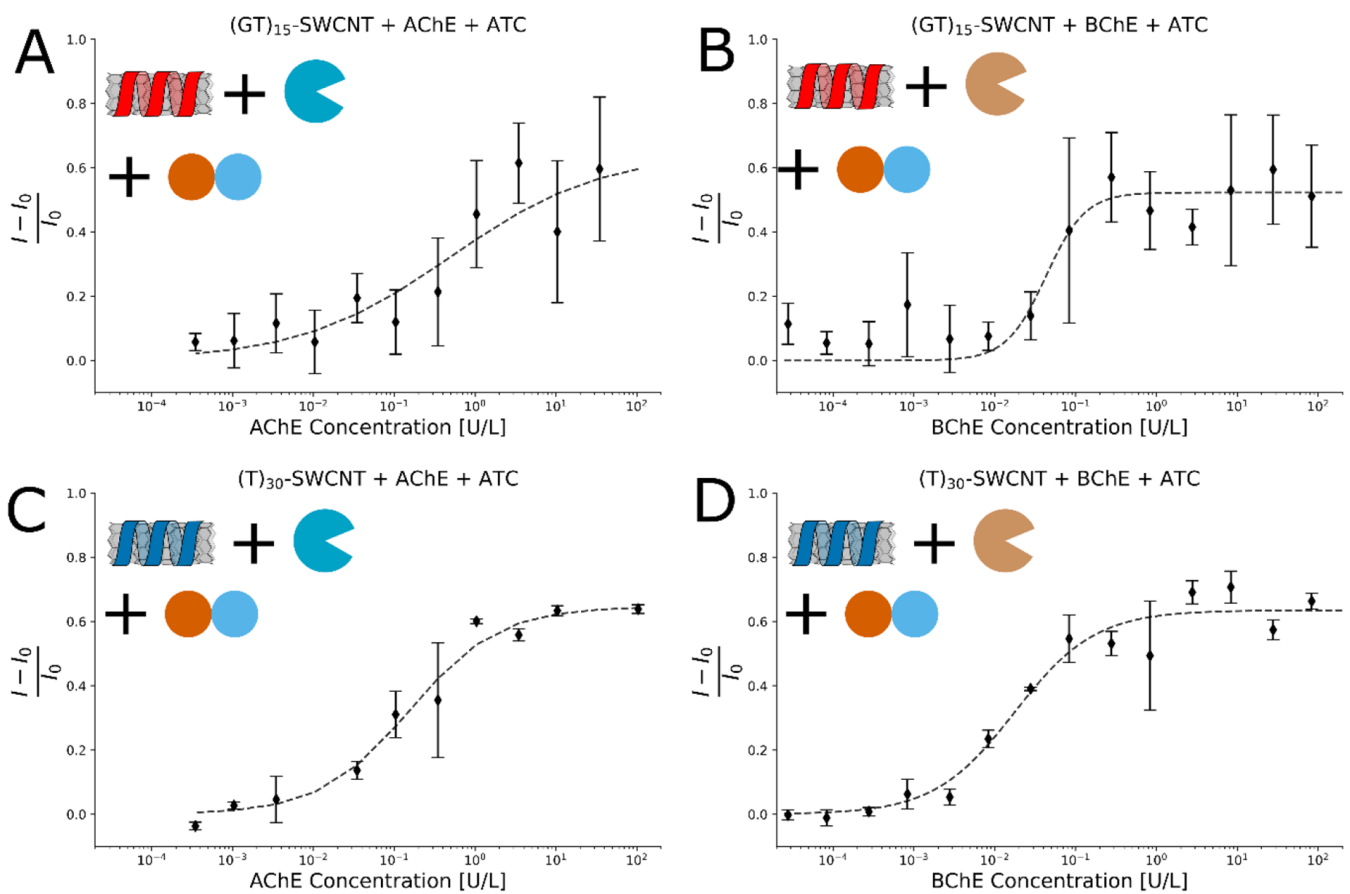
Fluorescence response of (GT)_15_-SWCNT and (T)_30_-SWCNT to different concentrations of AChE or BChE. (A) (GT)_15_-SWCNTs and AChE, (B) (GT)_15_-SWCNTs and BChE, (C) (T)_30_-SWCNTs and AChE, and (D) (T)_30_-SWCNTs and BChE. All error bars represent the standard deviation of experimental replicates (*n* = 3). Four parameter logistic regression functions were fitted to the results (dashed lines), and their parameters can be found in the SI. The inflection points are in the range of 10^–2^ to 10^–1^ U L^–1^.

The concentration-dependent normalized fluorescence response was fitted by a four parameter logistic regression with a zero baseline, resulting in a three-parameter fit,^45,46^, where *I*_0_ and *I* are the initial and final fluorescence intensity, respectively, *x* is the cholinesterase activity units concentration, β is a proportion constant corresponding to the normalized fluorescence response at saturation, *n* is a cooperativity factor, and *k* is the inflection point (Figure 2). We have chosen a fit function that saturates as ATC is expected to be depleted in these assay conditions for activity units larger than 1 U L^–1^, optimizing the response curve for physiological measurements. As such, the sigmoidal plots match the assay conditions and the amount of thiocholine present in the solution. Nevertheless, the assay conditions can be changed to fit the dynamic range of different ChE activity units. The fit parameters are summarized in the Supporting Information (Table S1).

**Figure 3:**
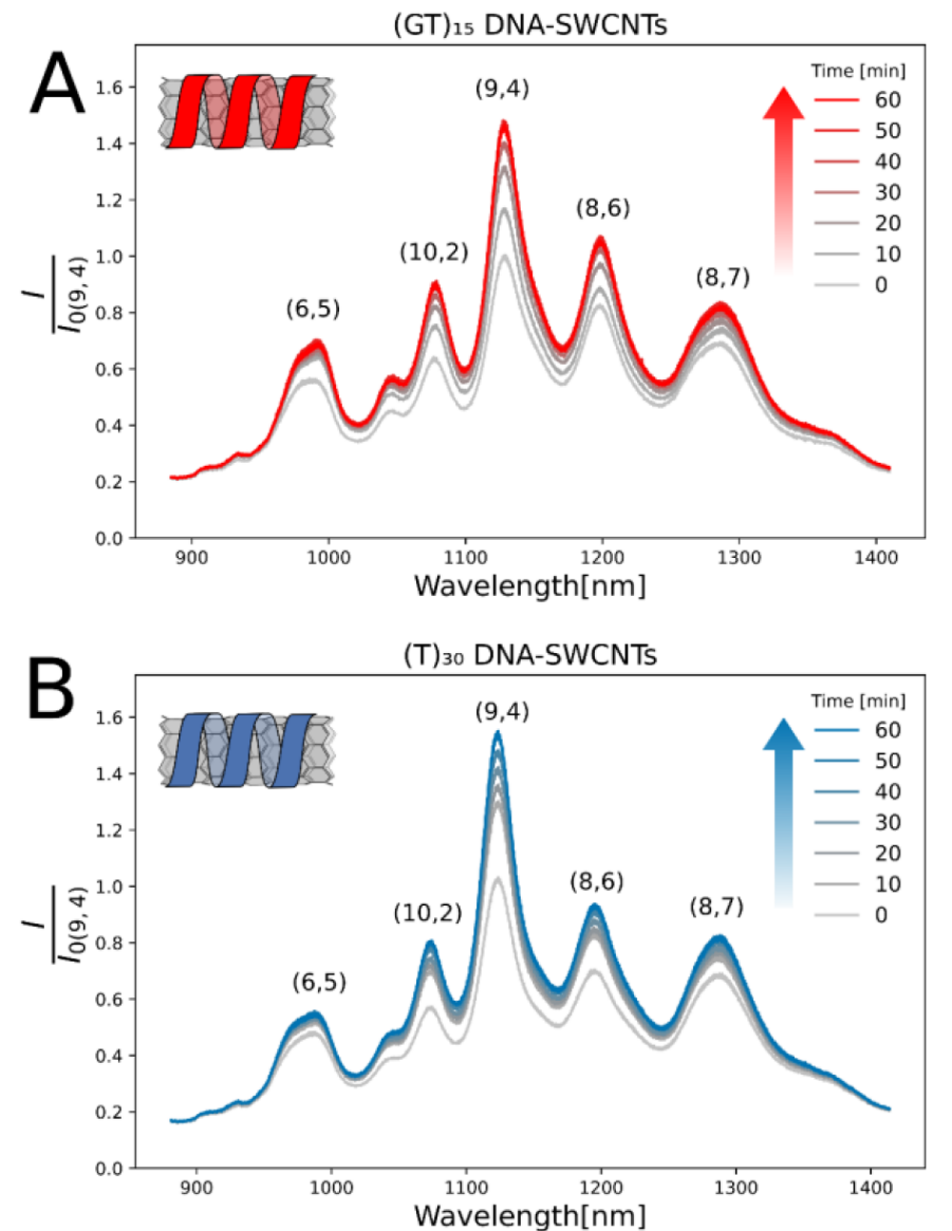
Time-dependent response of (GT)_15_-SWCNTs (A) and (T)_30_-SWCNTs (B) to 1 U L^–1^ AChE activity. The fluorescence response plateaus after about 1 h (Figure S4).

The *k* values obtained are in the range of 10^–2^ to 10^–1^ U L^–1^, and the response at saturation values, β, are in the range of 50–60%. Taking the experimental error into account, this fluorescence increase at saturation, resulting from the hydrolysis of 100 μM acetylthiocholine to thiocholine in the assay solution, is in good agreement with the ∼60–70% fluorescence increase seen when directly spiking the DNA-SWCNT sensor solutions with 100 μM thiocholine (Figure S3). The limit of detection was found to be in the 10^–2^ U L^–1^ range, mainly limited by the experimental error (Table S1), with values of 0.38 and 0.06 U L^–1^ for (GT)_15_-SWCNTs for AChE and BChE respectively, as well as values of 0.02 and 0.003 U L^–1^ for (T)_30_-SWCNTs for AChE and BChE respectively. The time-dependent response for 1 U L^–1^ cholinesterase activity in both DNA-SWCNT sensors is shown in Figure 3. The fluorescence response to cholinesterase activity equilibrates after approximately 1 h, showing an intensity increase for all measured DNA-SWCNT chiralities, most pronounced for the (9,4) chirality.

### Detecting Cholinesterase Inhibition

**Figure 4:**
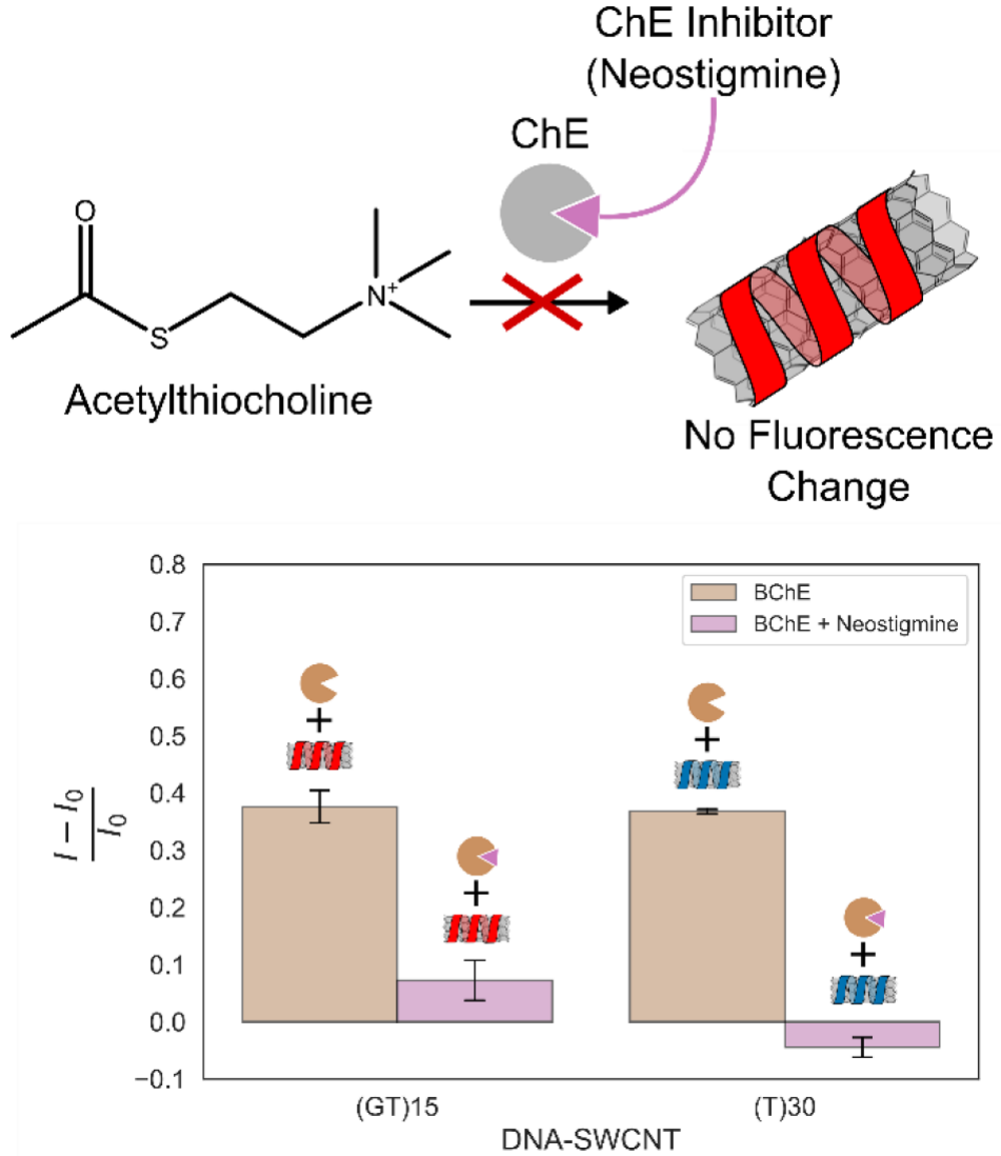
ChE inhibition by neostigmine illustration (top). Normalized fluorescence response of (GT)_15_-SWCNTs and (T)_30_-SWCNTs to inhibited and free BChE (bottom). A minimal fluorescence change is observed when a ChE inhibitor is present, compared to the significant response in the presence of the free ChE (*P*-value < 0.001 for both sensors). All error bars represent the standard deviation of experimental replicates (*n* = 3).

Neostigmine is an AChE inhibitor used for the treatment of myasthenia gravis.^67^ Neostigmine blocks the activity of cholinesterase enzymes, thus, it prevents the hydrolysis of acetylthiocholine to thiocholine (Figure 4, top). Since our DNA-SWCNT sensors respond to the product of the cholinesterase hydrolysis, they can also be used to infer whether the enzymes are inhibited. We exposed BChE to an excess amount of neostigmine in order to block ChE activity, and then added the substrate acetylthiocholine. The fluorescence intensity response was recorded after one hour, and compared to the uninhibited BChE (Figure 4, bottom). We found a significant difference in the fluorescence response between the inhibited and uninhibited cholinesterase conditions, where the fluorescence intensity in the uninhibited case was more than three times larger than the inhibited. This demonstration paves the way for the detection of cholinesterase inhibition, which is important for a wide range of applications, from screening drug candidates^3^ to detecting pesticides^68^ and nerve agents.^69,70^

### Detecting Serum Cholinesterase Activity

**Figure 5:**
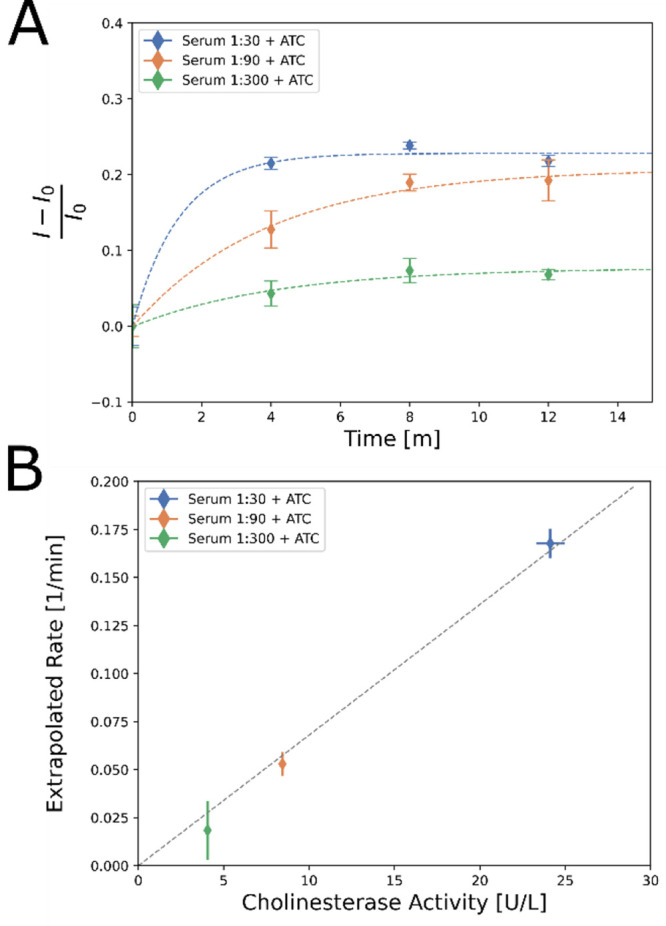
(A) Fluorescence intensity change of (GT)_15_-SWCNTs to various serum dilutions upon the addition of 450 μM acetylthiocholine. Error bars represent the standard deviation of experimental replicates (*n* = 3). (B) The extrapolated rate constant from the slope at *t* = 0 of the exponential fit in (A) plotted as a function of the cholinesterase activity rate measured in serum by the Ellman method. A line was added as a guide to the eye. Error bars represent the 95% confidence intervals of the fit function (*n* = 3).

To demonstrate the applicability of the sensors to real-world samples, we measured cholinesterase activity in diluted fetal bovine serum. Serum samples were diluted 1:30, 1:90, and 1:300 in PBS, and their cholinesterase activity was measured separately by the Ellman method. (GT)_15_-SWCNTs were incubated at 1 mg L^–1^ in diluted serum solution for two hours, after which 450 μM acetylthiocholine was added and the fluorescence intensity change was monitored for 12 min (Figure 5A). Each fluorescence intensity profile was fitted to an exponential function (Table S2). The slopes of the initial fluorescence intensity increase rate correlate well with the rate of production of the product of ChE activity measured by the Ellman assay (Figure 5B).

### Spatiotemporal Nanosensor Fluorescence Imaging

In order to demonstrate the fluorescence response at the level of individual DNA-SWCNT sensors, we imaged surface-adsorbed (GT)_15_-SWCNTs on a coverslip using HiLo microscopy^57^ and monitored the fluorescence response upon the addition of 450 μM of thiocholine. We resolved individual nanotubes and observed an increase in fluorescence intensity of 150–180% upon thiocholine addition (Figure S6 and Supporting Information, Video 1). Averaging the fluorescence intensity over the entire field of view (FOV) in response to the addition of thiocholine, we observed a 70% increase compared to the initial fluorescence intensity The higher response for individual SWCNT sensors, compared to the full FOV, is expected, since in the latter case, the areas without SWCNTs are taken into account in the calculation, masking the signal of the individual SWCNTs. This experiment demonstrates the potential for spatiotemporally resolving ChE activity in the near-infrared optical range using the DNA-SWCNT sensor platform.

### Enzyme Activity Imaging

**Figure 6:**
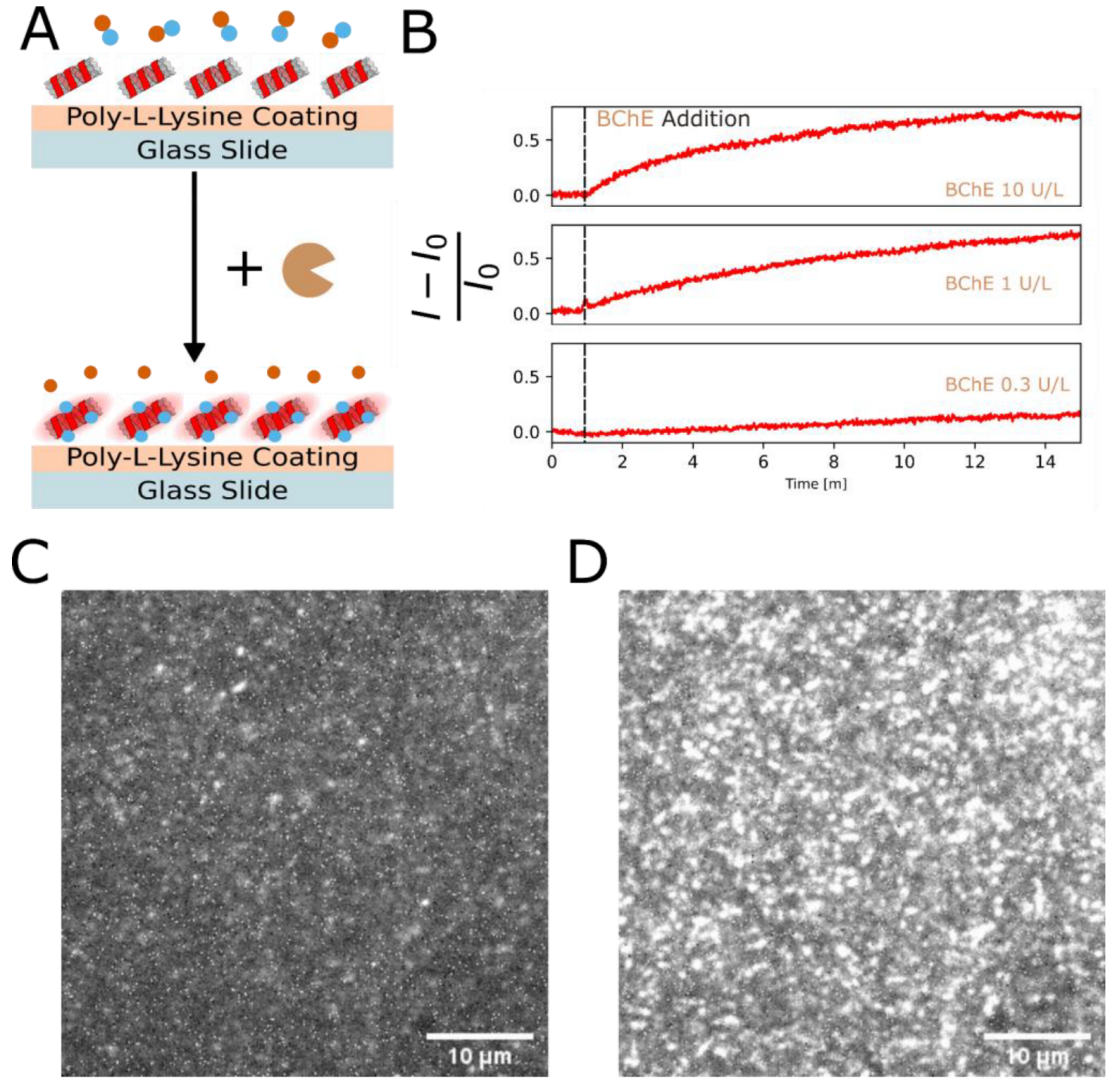
(A) Schematic diagram of the enzyme activity imaging experiment. (B) Full field of view fluorescence intensity time-traces showing the response to various cholinesterase concentrations added to SWCNTs with 450 μM acetylthiocholine. Images of (GT)_15_-SWCNTs before (C) and after (D) the addition of butyrylcholinesterase. Images were extracted from a movie taken with a 100× objective. Scale bars represent 10 μm.

We then proceeded to measure the enzyme activity directly. SWCNTs in 450 μM acetylthiocholine were spiked with 5 μL of different BChE concentrations, and the fluorescence response was monitored (Figure 6, Supporting Information, Video 2). Analyzing the entire FOV, there is a clear fluorescence intensity increase in all samples, for which the kinetics depends on the enzyme concentration. The fluorescence intensity increases by up to 70%, in agreement with the data presented in Figures S3 and S6.

## Conclusions

We demonstrated two near-IR fluorescent single-walled carbon nanotube sensors for the measurement of cholinesterase activity and inhibition by screening five different DNA-SWCNTs candidates, and identifying two promising sensors, (GT)_15_-SWCNTs and (T)_30_-SWCNTs. These sensors showed a large and selective response toward thiocholine, the ChE hydrolysis product of acetylthiocholine, and almost no response to the substrate acetylthiocholine, the byproduct acetic acid, nor to the ChE inhibitor. The sensors fluorescence response was concentration-dependent, yielding LOD values in the range of 10^–3^–10^–1^ U L^–1^, well within the relevant concentration range for physiological measurements, and was demonstrated in serum samples. We demonstrated the sensors ability to infer the presence of cholinesterase inhibitors, manifested in a diminishing fluorescence response compared to the free enzyme, by a factor of more than 3. Lastly, we imaged immobilized DNA-SWCNTs and observed an immediate fluorescence intensity increase in response to thiocholine and ChE activity at the level of individual nanosensors.

In summary, our DNA-SWCNT sensors for ChE activity and inhibition can be used in numerous settings where a rapid, in situ, reading of ChE activity is required, such as for sensing pesticide residues or for surgical guidance based on cancer-associated cholinesterase activity difference. Our work enables numerous applications using the option for spatiotemporal information of ChE activity and inhibition with our sensor platform and optical signal transduction in the biological transparency window.

## Supporting Information Available

Video S1 (AVI)
Video S2 (AVI)
Excitation emission spectra of DNA-SWCNTs, ssDNA library fluorescence responses, response of DNA-SWCNT sensors to various small molecules, time-dependent fluorescence response to 1 U L^–1^ BChE, response of DNA-SWCNTs to various serum dilutions, thiocholine imaging experiment, four parameter logistic regression fit parameters, and exponential fit parameters (PDF)

## Supplementary Material

ac2c02471_si_001.avi

ac2c02471_si_002.avi
