# Supplementary material for: Monitoring the Activity and Inhibition of Cholinesterase Enzymes using Single-Walled Carbon Nanotube Fluorescent Sensors
Source: Anal Chem. 2022 Oct 7;94(41):14223–31. doi: 10.1021/acs.analchem.2c02471 (PMC9583068; doi:10.1021/acs.analchem.2c02471)
Supplement: Supplementary file 3 — ac2c02471_si_003.pdf [file ac2c02471_si_003.pdf]

Supporting Information

Monitoring the Activity and Inhibition of Cholinesterase Enzymes using Single-Walled Carbon Nanotube Fluorescent Sensors

Dan Loewenthal[1,2], Dotan Kamber[3], Gili Bisker[3,4,5,6]*

[1] School of Chemistry, Faculty of Exact Sciences, Tel-Aviv University, Tel Aviv 6997801, Israel

[2] Department of Analytical Chemistry, Israel Institute for Biological Research, Ness-Ziona 7410001, Israel

[3] Department of Biomedical Engineering, Faculty of Engineering, Tel-Aviv University, Tel Aviv 6997801, Israel

[4] The Center for Physics and Chemistry of Living Systems, Tel-Aviv University, Tel Aviv 6997801, Israel

[5] Center for Nanoscience and Nanotechnology, Tel-Aviv University, Tel Aviv 6997801, Israel

[6] Center for Light Matter Interaction, Tel-Aviv University, Tel Aviv 6997801, Israel

*Corresponding author

Email: bisker@tauex.tau.ac.il

TABLE OF CONTENTS

Figure S1 – Excitation emission spectra of DNA-SWCNTs

Figure S2 – ssDNA library fluorescence responses

Figure S3 – Response of DNA-SWCNT sensors to various small molecules

Figure S4 – Time-dependent fluorescence intensity of DNA-SWCNTs in response to 1 U L^{-1} BChE

Figure S5 – Response of DNA-SWCNTs to various serum dilutions

Figure S6 – Thiocholine imaging experiment

Table S1 – Four parameter logistic regression fit parameters

Table S2 – Exponential fit parameters

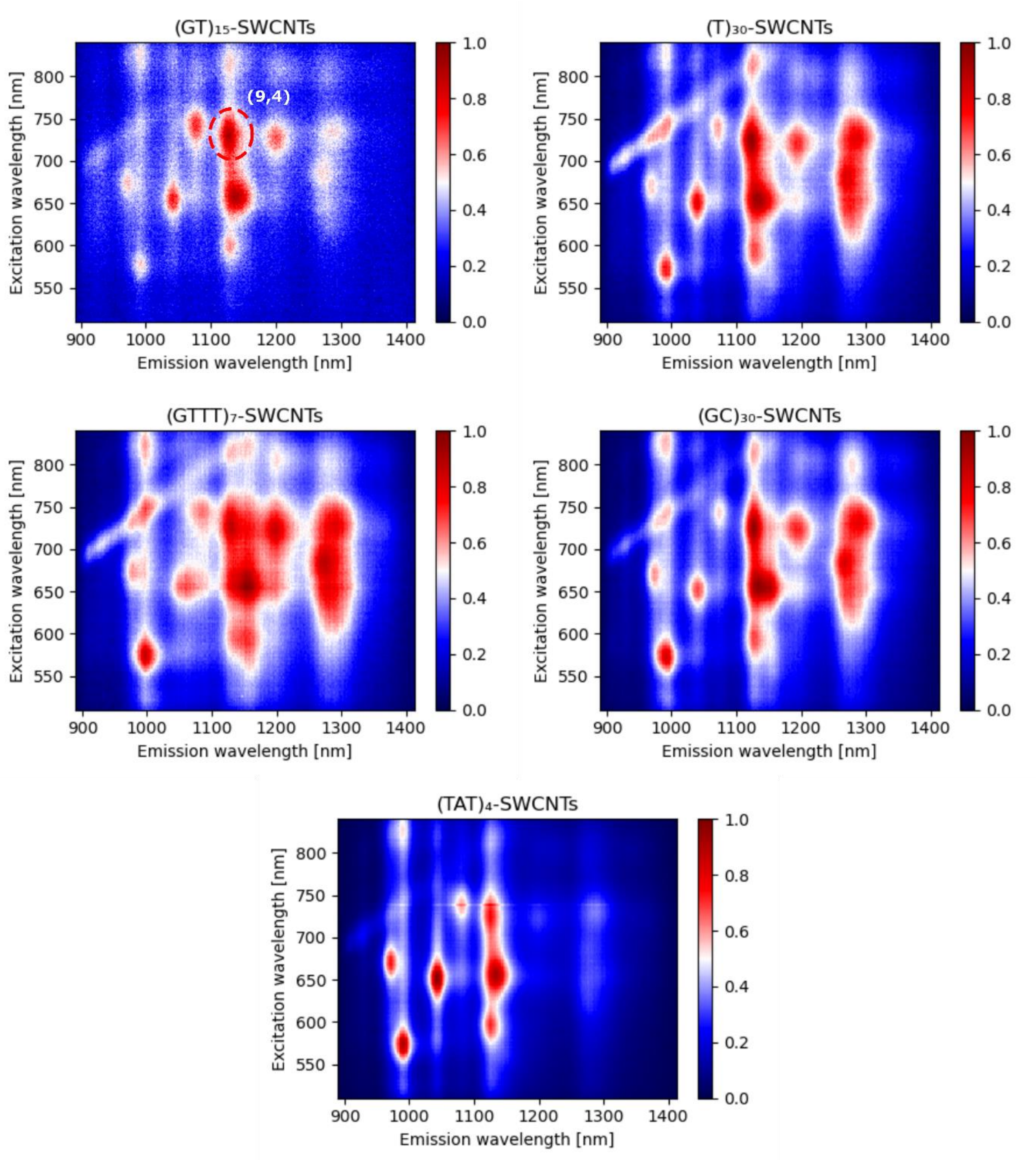

Figure S1. Excitation-emission fluorescence spectra of screened DNA-SWCNTs. The (9,4) chirality is highlighted for (GT)$_{15}$-SWCNTs.

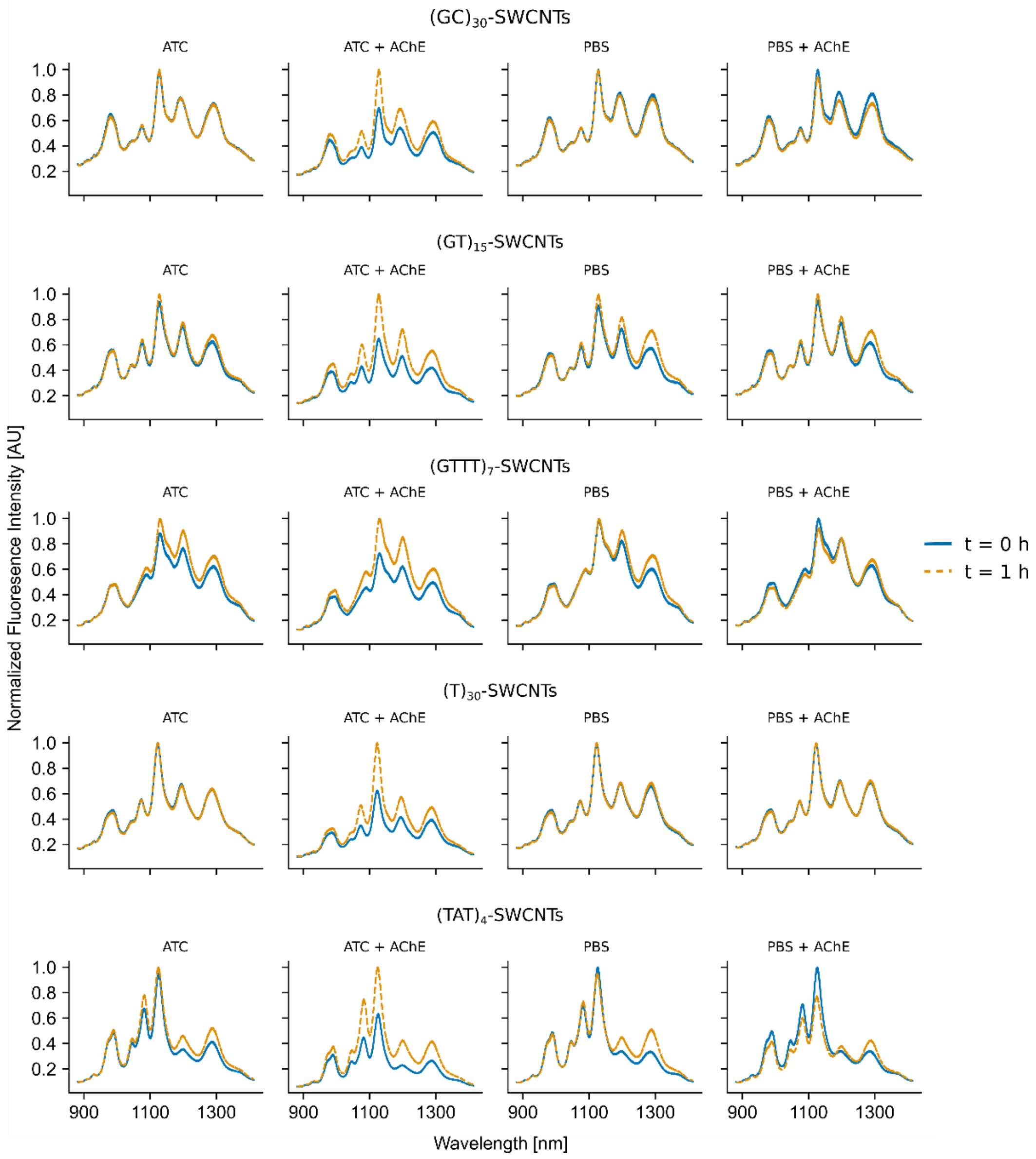

Figure S2. DNA-SWCNTs screening results. Each row shows a different sensor, and each column shows a different analyte addition (from left to right): acetylthiocholine (ATC), ATC followed by acetylcholinesterase (AChE), PBS, PBS followed by AChE. The blue lines are the fluorescence intensity at $t = 0$ upon the addition of the analyte, and the orange lines are the fluorescence intensity at $t = 1\ h$. Each spectrum contains the average data of three different experiments. The chosen sensors, $(GT)_{15}$-SWCNT and $(T)_{30}$-SWCNT, show no response to PBS, AChE alone, nor acetylthiocholine alone, but show a fluorescence intensity increase in response to AChE in the presence of acetylthiocholine.

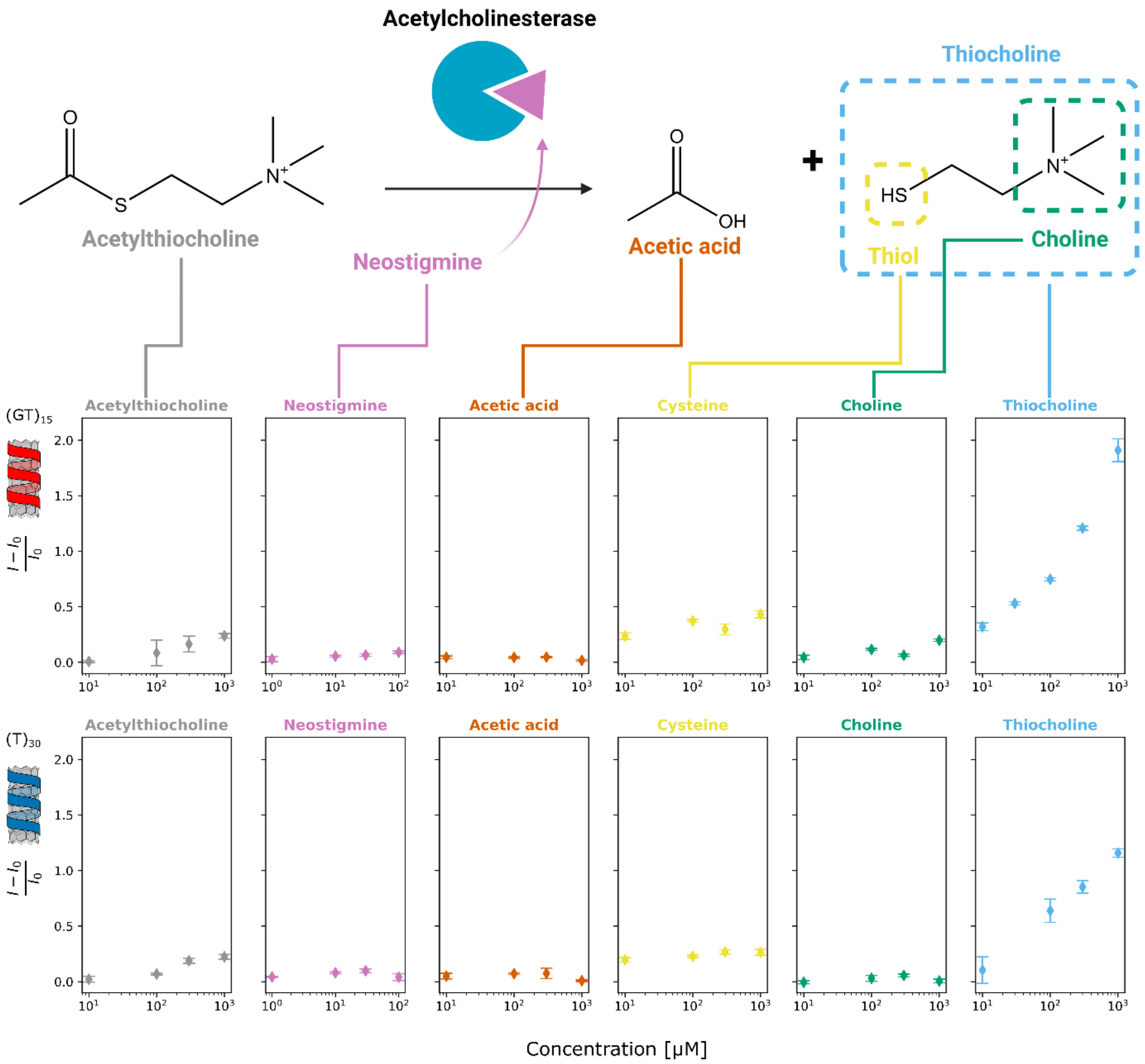

Figure S3. Relevant small molecules for the AChE-mediated hydrolysis of acetylthiocholine to acetic acid and thiocholine with\without the presence of an inhibitor (top row). Normalized fluorescence response of $(GT)_{15}$-SWCNTs (middle row) and $(T)_{30}$-SWCNTs (bottom row) nanosensors to small molecules. DNA-SWCNTs exhibit a large and concentration-dependent response for thiocholine, whereas other molecules induce a small to negligible response All error bars represent the standard deviation of experimental replicates ($n = 3$).

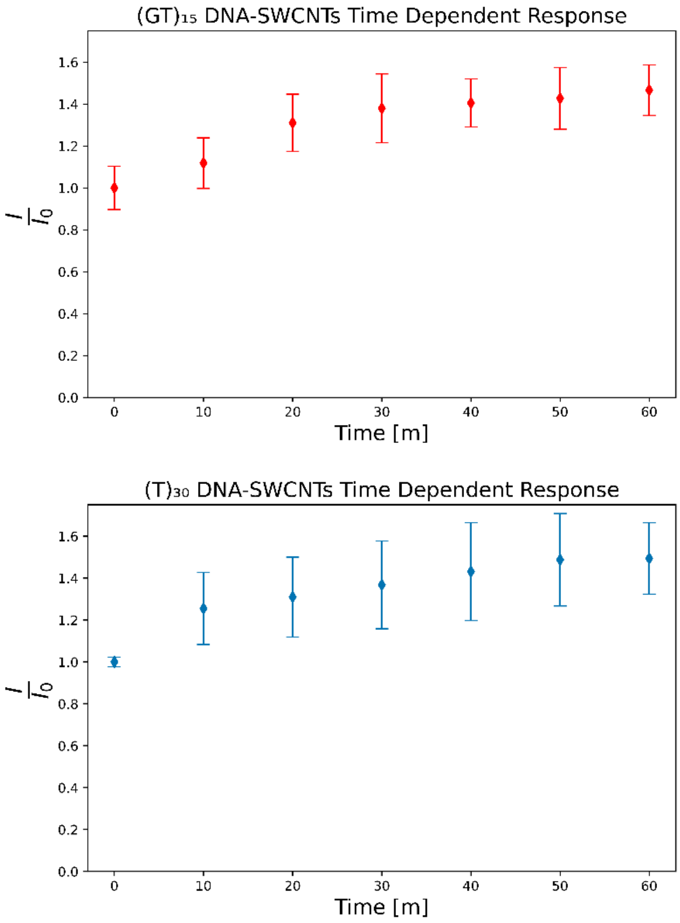

Figure S4. Time-dependent fluorescence intensity for the (9,4) chirality peak of $(GT)_{15}$-SWCNTs (top) and $(T)_{30}$-SWCNTs (bottom) in response to 1 U L^{-1} butyrylcholinesterase in the presence of acetylthiocholine. All error bars represent the standard deviation of experimental replicates ($n = 3$).

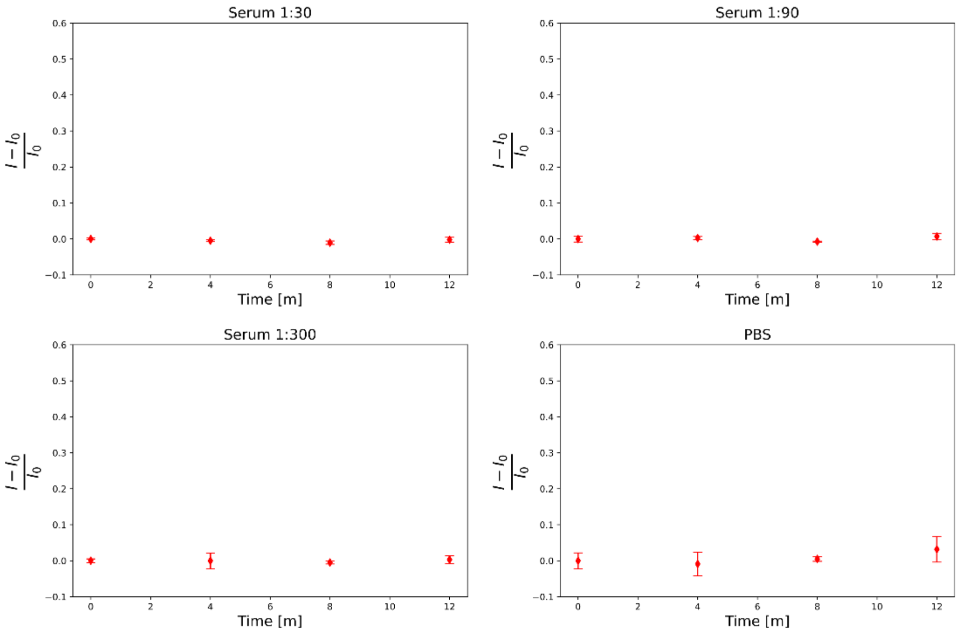

Figure S5. Time-dependent fluorescence intensity for the (9,4) chirality peak of $(GT)_{15}$-SWCNTs in various serum dilutions after two hours of incubation. There was little to no change in the fluorescence intensity of the SWCNTs during the serum cholinesterase activity assay.

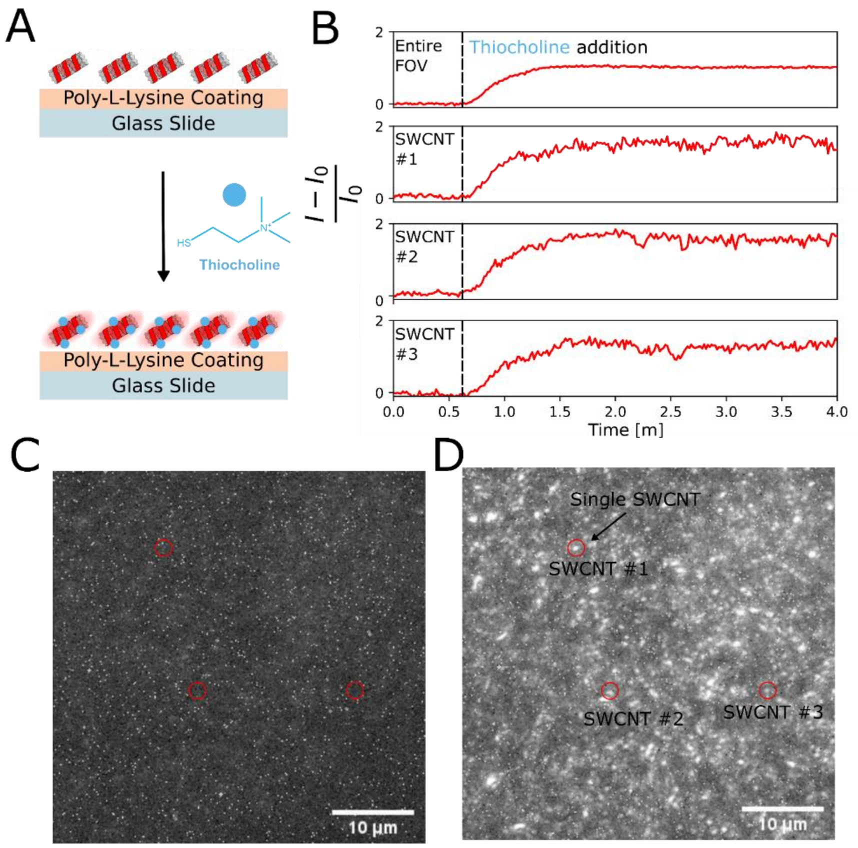

Figure S6. (A) Schematic diagram of the thiocholine imaging experiment. $(GT)_{15}$-SWCNTs were immobilized on a Poly-L-Lysine treated glass slide, to which thiocholine solution was added. (B) Fluorescence intensity response of the entire field of view (FOV) of single $(GT)_{15}$-SWCNTs (bottom graphs) during addition, showing a clear increase in fluorescence intensity upon thiocholine addition. Images of $(GT)_{15}$-SWCNTs before (C) and after (D) the addition of thiocholine. Images were extracted from a movie taken with a 100 × objective. Scale bars represent 10 µm.

	$(GT)_{15}$ -SWCNT + AChE + ATC	$(GT)_{15}$ -SWCNT + BChE + ATC	$(T)_{30}$ -SWCNT + AChE + ATC	$(T)_{30}$ -SWCNT + BChE + ATC
β	0.6 ± 0.33	0.5 ± 0.07	0.6 ± 0.07	0.6 ± 0.03
$k\ [U \cdot L^{-1}]$	$(4.6 \pm 13.42) \times 10^{-1}$	$(4.3 \pm 2.98) \times 10^{-2}$	$(1.5 \pm 0.82) \times 10^{-1}$	$(1.8 \pm 0.70) \times 10^{-2}$
n	0.50 ± 0.42	1.8 ± 1.92	0.8 ± 0.30	0.9 ± 0.26
$LOD\ [U \cdot L^{-1}]$	0.38	0.06	0.02	0.003

Table S1. Four parameter logistic regression fit parameters, with a zero baseline. Values were extracted by curve fitting using MATLAB and are displayed $\pm$ their 95% confidence interval. $\frac{I-I_0}{I_0} = \beta \frac{x^n}{x^n+k^n}$, where I is the final fluorescence intensity, I_0 is the initial fluorescence intensity, β is a proportion constant equal to the intensity at saturation, k is the inflection point, n is a cooperativity factor, and x is cholinesterase concentration.

	**$(GT)_{15}$-SWCNT + 1:30 FBS + 450 μM ATC**	**$(GT)_{15}$-SWCNT + 1:90 FBS + 450 μM ATC**	**$(GT)_{15}$-SWCNT + 1:300 FBS + 450 μM ATC**
a	0.22 ± 0.02	0.21 ± 0.04	0.08 ± 0.04
b [min^{-1}]	0.7 ± 0.4	0.2 ± 0.1	0.2 ± 0.3

Table S2. Exponential function fit parameters. Values were extracted by curve fitting to an exponential function using MATLAB and are displayed $\pm$ their 95% confidence interval. $\frac{I-I_0}{I_0} = a \cdot (1 - e^{-b \cdot t})$, where I is the final fluorescence intensity, I_0 is the intensity of the relevant control serum sample without ATC addition, b is the rate constant, a is the saturation value at the long-time limit, and t is the time since ATC addition in minutes.
